# Does Change in Physical Activity During the Initial Phase of the COVID-19 Pandemic Predict Psychological Symptoms in Physically Active Adults? A Six-Month Longitudinal Study

**DOI:** 10.3389/ijph.2022.1604528

**Published:** 2022-06-08

**Authors:** Audun Havnen, Linda Ernstsen

**Affiliations:** ^1^ Department of Psychology, Norwegian University of Science and Technology, Trondheim, Norway; ^2^ St. Olav’s University Hospital, Trondheim, Norway; ^3^ Department of Public Health and Nursing, Norwegian University of Science and Technology, Trondheim, Norway

**Keywords:** anxiety, mental health, COVID-19, physical activity, depression

## Abstract

**Objectives:** The current study aimed to investigate if change in physical activity during the initial phase of the COVID-19 pandemic predicted severity of anxiety and depression symptoms 6 months later in physically active adults.

**Methods:** A total of 855 respondents (32.6% women) completed the Hospital Anxiety and Depression Scale (HADS) at two time points and reported change in physical activity habits in the first 3 months of the COVID-19 lockdown in Norway.

**Results:** Women had higher prevalence rates than men for both anxiety and depression symptoms in the Unchanged, Increased and Decreased physical activity (PA) subgroups. Women and men who reported Increased PA at baseline were associated with increased risk for anxiety symptoms at time 2. Increased PA was associated with higher risk for depression at time 2 for women, but not for men.

**Conclusion:** The results indicate that the COVID-19 pandemic is associated with deterioration in mental health also for physically active adults.

## Introduction

The Novel Coronavirus Disease 19 (COVID-19) was declared a public health emergency by the World Health Organization [1] on 30 January 2020, and has had a large effect on people’s lives globally subsequently. The large death toll due to the coronavirus (SARS-Cov-2 and related mutant viruses) has impacted families worldwide and social restriction rules have had a detrimental impact on social network and everyday life. The global economy has been greatly affected and many people have lost their income [2]. The pandemic situation thus represents a high degree of uncertainty about the future and fear of contracting the coronavirus [3].

During the initial phase of the COVID-19 pandemic studies reported a marked deterioration in mental health [4, 5]. It is well documented that life stressors and economic hardship may increase the risk for depression [6] and anxiety [7], and hence it was argued that the pandemic situation itself represented a stressor in people’s lives that may lead to mental health challenges [8]. In addition, social distancing measures, like quarantine and lockdown, were also found to be related to symptoms of post-traumatic stress, anxiety and depression [9]. Longitudinal studies showed an increase in symptoms of anxiety and depression in the general population compared to before the COVID-19 pandemic, with women and younger people more vulnerable to deterioration in mental health [10]. The combination of the uncertainty experienced during the pandemic situation added to strict mitigation strategies thus represent parallel pathways that may negatively impact mental health.

Physical activity has been demonstrated to improve mental health status and protect against depression and anxiety in both non-clinical [11] and clinical populations [12]. Physical activity is also beneficial for physical health and protects against a range of diseases [13]. The World Health Organization has recommended that adults undertake 150–300 min of moderate intensity or 75–150 min of vigorous intensity physical activity per week [14]. During the COVID-19 pandemic studies show a decrease in physical activity [15–17]. This decrease has been associated with poorer mental health, and individuals who maintain higher levels of physical activity have lower risk for both depressive symptoms and anxiety symptoms [18, 19].

The empirical evidence showing that physical activity reduced risk of depression during COVID-19 suggests that remaining physically active may protect persons from negative emotional outcome that is related to the pandemic [18]. This is especially important as researchers have suggested an increase in untreated mental illness during the pandemic as psychiatric referrals dropped [20]. Physical activity may be promoted as a feasible and cost-effectiveness measure to improve mental health, both in high-income countries with reduced referral rates as well as in low-income countries with limited access to mental health care [18].

Many of the early studies during the COVID-19 pandemic were cross-sectional and relied on having the participants retrospectively report changes in physical activity from the pre-pandemic period to during the COVID-19. Such early studies were limited by the lack of follow-up assessment to measure long-term outcomes. However, studies with prospective longitudinal designs are starting to emerge, and many of the studies show that physical activity is associated with lower anxiety and depressive symptoms [21, 22]. However, many of the published studies have had short follow-up periods, thus studies with longer time spans are warranted in order to detect changes in mental health over time.

Although studies have investigated the role of physical activity in the general population, such findings may not be generalizable to populations of highly physically active individuals. This is partly due to the fact that the general public tend to exercise less than the recommendations by the WHO [23]. This is illustrated further by evidence showing that many recreational athletes increased weekly exercise during periods with lockdown restrictions [24]. Although studies indicate that physical activity is a protective factor for mental health in the general population, an important question is if persons who are highly active on a regular basis are not prone to emotional consequences during the COVID-19 pandemic due to the protective effect of physical activity, or if also these individuals may suffer negative emotional effects. There is evidence to suggest that mental health was negatively affected during home confinement among elite athletes [25] and young athletes [26]. These findings suggest that even persons who have high levels of physical activity may experience negative emotional reactions during the pandemic situation. Our findings from a relatively physically active cohort of members of a Norwegian endurance sports organization showed an association between self-reported change in physical activity, mental health and sleep disturbances during the initial phase of the pandemic [27, 28]. These studies reported a lower prevalence of clinically relevant anxiety and depression symptoms in the initial phase of the lockdown compared to the general population. However, if mental health in physically active adults changed during the COVID-19 pandemic is unknown. It is therefore a need for studies with longitudinal designs that investigate the long-term association between change in physical activity and mental health during the COVID-19 pandemic among highly active adults.

The present paper describes results from a longitudinal study of members of an endurance sports organization during the COVID-19 pandemic. The study aimed to assess if change in physical activity during the initial phase of the pandemic was related to change in symptoms of anxiety and depression over a period of 6 months.

## Methods

### Participants

This study reports outcome from the first and the second wave from an ongoing longitudinal survey of members of an endurance sports organization. Invitations to an online survey were sent by e-mail to 6,766 persons (men = 75%). Respondents had to consent electronically to participate. Data was collected in two waves; time 1 in June 2020 and time 2 in January 2021. Time 1 was a period with lockdown in the Norwegian society. During this period, universities, schools and kindergartens were closed, public transportation should be avoided and employees had to work from home. Time 2 was conducted 6 months later during which period Norwegian authorities implemented more strict social distancing regulations, after a period with low levels of social distancing measures. From January 2021, social distancing regulations included lectures at universities to be completely online, recommendations of working from home offices, maximum number of five guests in private households, restrictions in number of customers in shops, and no alcohol serving in restaurants. A total of 1,317 persons participated at time 1 and of these 855 (32.6% women) also completed assessment at time 2 6 months later. Those lost to follow-up (*n* = 462) were significantly younger than those who participated at both time-points, but the samples did not differ on any other background variables (see Supplementary Table S1). The study was approved by the Regional Committee for Ethics in Medical Research in Norway and the Norwegian Centre for Research Data.

### Measures

#### Demographics

Participants provided information about age, gender, education, alcohol use, long-term limiting illness (mental or physical), history of mental disorder, and current use of psychotropic drugs.

#### Physical Activity

Physical activity (PA) was assessed in terms of frequency, intensity and duration. Frequency was stated as “How often do you exercise?” with five response options (never, less than once a week, once a week, two to three times a week, and four or more times a week). Intensity of exercise was stated as, “How hard do you exercise?” with three response options (“no sweat or heavy breath,” “heavy breath and sweat,” and “push myself to exhaustion”). For duration of exercise respondents stated one out of four options (“<15 min,” “between 15 and 30 min,” “between 30 and 60 min,” and “>60 min”). The PA questions have been validated against objective measures of PA and the International Physical Activity Questionnaire [29] Physical Activity Index (PA-I) was calculated in accordance with the procedure described in Nes et al. [30].

We applied the WHO guidelines for recommended level of weekly physical activity [13] to assess level of weekly exercise. The guidelines recommend that adults undertake 150–300 min of moderate intensity or 75–150 min of vigorous intensity physical activity per week. At timepoint 1 participants were asked if they had changed the amount of weekly physical activity during the COVID-19 Norwegian lockdown (March 2020 to June 2020), with the nominal response options “unchanged,” “increased” or “decreased PA.”

#### Anxiety and Depression

The Hospital Anxiety and Depression Scale [HADS; [31] was used to measure symptoms of anxiety and depression. The HADS contains 14 items, of which 7 assess anxiety symptoms (HADS-A) and 7 assess depressive symptoms (HADS-D). For respondents who had completed 6 items on HADS-A time 1 (*n* = 8), HADS-A time 2 (*n* = 12), HADS-D time 1 (*n* = 10) and HADS-D time 2 (*n* = 4), the sum was multiplied by 7/6 to calculate the total score, an approach that has been used elsewhere [32]. A cut-off of 8 was used to determine symptom severity indicative of a clinical symptom level. The HADS is a reliable and valid measure of symptom level for both anxiety and depression in patient populations and in the general population, and he Norwegian version of the HADS has demonstrated good psychometric properties [33].

### Statistical Analysis

Changes in mean scores over time were tested with paired samples t-tests. Cohen’s *d* was calculated from the t-statistics as *d* = M/SD, where M refers to the mean difference and SD refers to the standard deviation of the mean difference. Binary logistic regression was used to measure the relationship between change in PA with anxiety and depression symptoms separately, adjusting for age, sex, education, alcohol use, current psychotropic drug use, limiting long-term illness (physical or mental), history of mental disorder, and baseline symptom level. In analysis with anxiety disorder (HADS-A ≥ 8) as dependent variable, the model was adjusted for baseline HADS-A score, and opposite, for depression (HADS-D ≥ 8) as the dependent variable, the model was adjusted for baseline HADS-D score. An interaction term (sex∗change in PA) was included to assess if sex moderated the association between change in PA and mental health. The interaction was statistically significant for analysis with depression symptoms (*p* < 0.05), but not for anxiety symptoms (*p* = 0.80). Multiple linear regression analysis with HADS-A and HADS-D total score as dependent variables in two separate analyses were conducted using the same control variables as described above. Since the interaction term was significant for depression symptoms and the fact that sex differences are common with respect to mental health problems, all analyses were stratified by sex. Statistical analyses were conducted in IBM SPSS version 27.

## Results

### Sex Differences in Prevalence of Anxiety and Depression Symptoms

**FIGURE 1:**
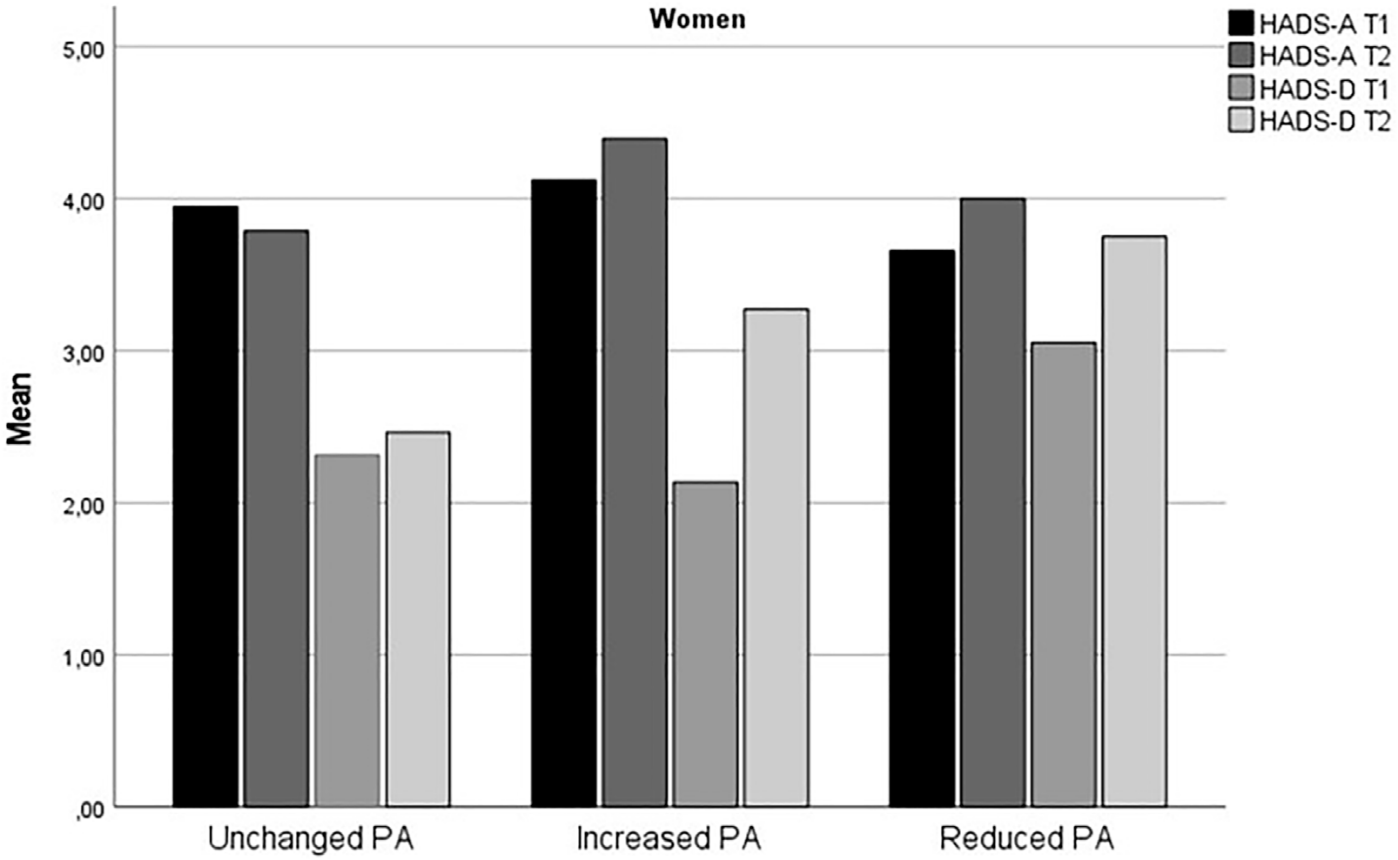
Change in anxiety and depression symptoms from time 1 to time 2 for women (The fitness and mental health study, Norway, 2020–2021).

**FIGURE 2:**
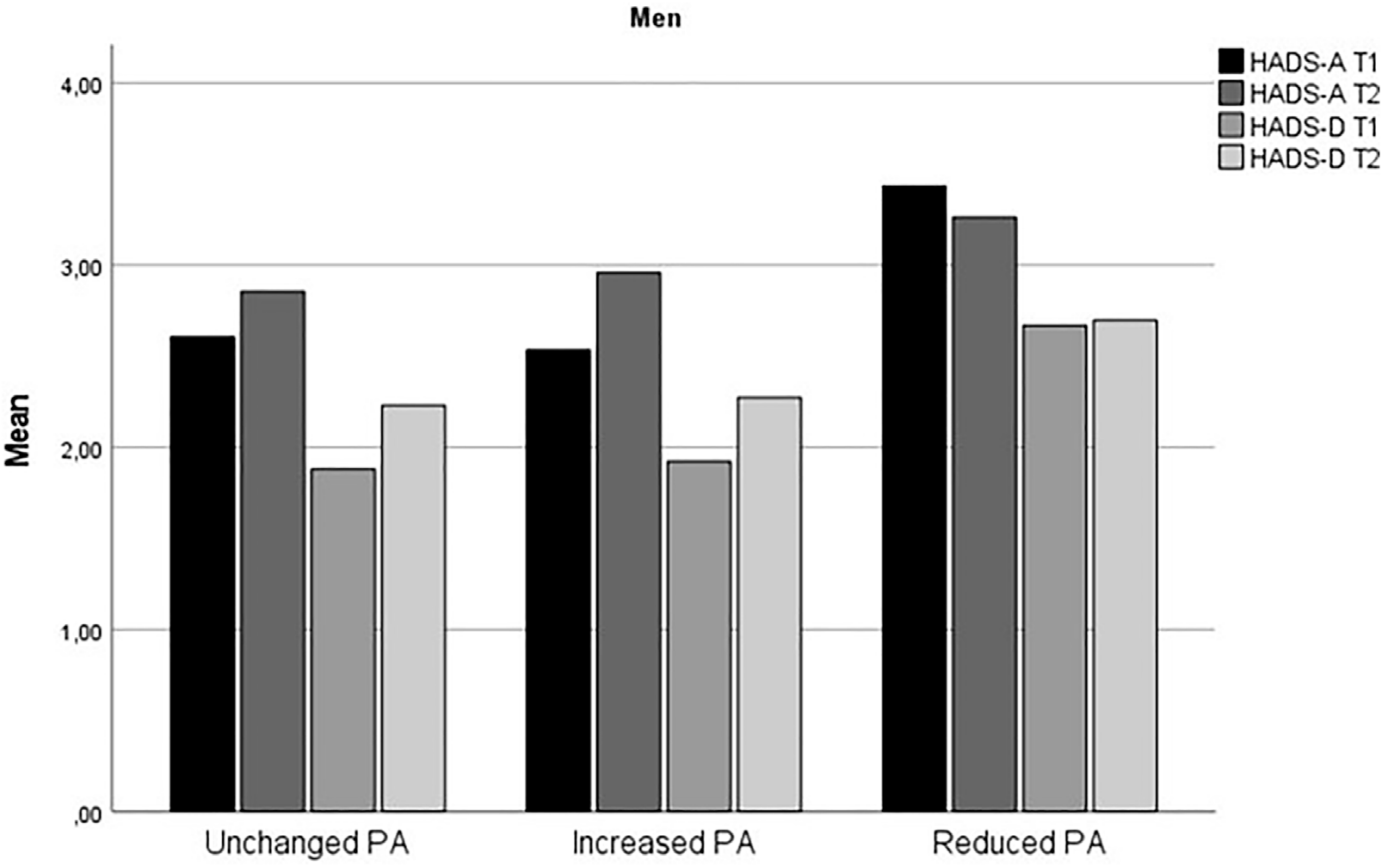
Change in anxiety and depression symptoms from time 1 to time 2 for men (The fitness and mental health study, Norway, 2020–2021).

Table 1 summarizes the demographic and other basic information of the respondents, stratified by sex and change in PA. Change in HADS-A and HADS-D from time 1 to time 2 for stratified by sex and level of physical activity is displayed in Figures 1, 2.

**TABLE 1:** Demographics stratified by sex and change in physical activity level at time 1 (The fitness and mental health study, Norway, 2020–2021).

	Women (*n* = 279)			Men (*n* = 576)		
	Unchanged PA	Increased PA	Reduced PA	Unchanged PA	Increased PA	Reduced PA
	*n* (%)	*n* (%)	*n* (%)	*n* (%)	*n* (%)	*n* (%)
Age, years (mean ± SD)	47.41 (9.98)	45.00 (10.52)	47.32 (10.18)	52.78 (11.71)	47.57 (9.78)	48.45 (11.14)
Education						
Primary, secondary, tertiary	14 (8.0)	4 (6.1)	3 (7.9)	30 (7.8)	8 (7.0)	8 (10.5)
University/college <4 years	43 (24.6)	16 (24.2)	16 (42.1)	147 (38.1)	40 (35.1)	22 (28.9)
University/college >4 years	118 (67.4)	46 (69.7)	19 (50.0)	209 (54.1)	66 (57.9)	46 (60.5)
Lifetime history of mental disorder						
Yes	36 (20.6)	15 (22.7)	12 (31.6)	21 (5.4)	9 (7.9)	6 (7.9)
No	139 (79.4)	51 (77.3)	26 (68.4)	365 (94.6)	105 (92.1)	70 (92.1)
Limiting long term illness						
Yes	32 (18.3)	9 (13.6)	9 (23.7)	27 (7.0)	10 (8.8)	4 (5.3)
No	143 (81.7)	57 (86.4)	29 (76.3)	359 (93.0)	104 (91.2)	72 (94.7)
Current psychotropic drug						
Yes	9 (5.1)	2 (3.0)	2 (5.3)	6 (1.6)	1 (0.9)	0
No	166 (94.9)	64 (97.0)	36 (94.7)	380 (98.4)	110 (99.1)	76 (100.0)
Alcohol use						
Once a month or less	55 (31.4)	24 (36.4)	12 (31.6)	114 (29.5)	30 (26.3)	20 (26.3)
2–4 times per month	69 (39.4)	21 (31.8)	20 (52.6)	134 (34.7)	38 (33.3)	28 (36.8)
2–3 times per week	42 (24.0)	18 (27.3)	5 (13.2)	121 (31.3)	33 (28.9)	22 (28.9)
4 times per week or more	9 (5.1)	3 (4.5)	1 (2.6)	17 (4.4)	13 (11.4)	6 (7.9)
Current weekly physical activity						
Once a week or less	1 (0.6)	0	3 (7.9)	3 (0.8)	0	3 (3.9)
2–3 times a week	33 (18.9)	9 (13.6)	16 (42.1)	100 (25.9)	16 (14.0)	36 (47.4)
About daily	140 (80.0)	57 (86.4)	19 (50.0)	282 (73.1)	98 (86.0)	37 (48.7)
HADS-A T1 (mean ± SD)	3.93 (3.70)	4.12 (3.59)	3.66 (3.48)	2.60 (2.63)	2.54 (2.26)	3.43 (3.18)
HADS-A ≥ 8 T1						
Yes	26 (14.9)	6 (9.1)	9 (23.7)	21 (5.4)	5 (4.4)	12 (15.8)
No	149 (85.1)	60 (90.9)	29 (76.3)	365 (94.6)	109 (95.6)	64 (84.2)
HADS-A T2 (mean ± SD)	3.79 (3.59)	4.39 (3.61)	4.00 (3.48)	2.86 (2.91)	2.96 (2.96)	3.26 (2.90)
HADS-A ≥ 8 T2						
Yes	23 (13.1)	9 (13.6)	7 (18.4)	22 (5.7)	10 (8.8)	9 (11.8)
No	152 (86.9)	57 (86.4)	31 (81.6)	364 (94.3)	104 (91.2)	67 (88.2)
HADS-D T1 (mean ± SD)	2.31 (2.44)	2.14 (2.20)	3.05 (2.72)	1.88 (2.20)	1.92 (2.00)	2.63 (2.58)
HADS-D ≥ 8 T1						
Yes	11 (6.3)	3 (4.5)	4 (10.5)	11 (2.8)	2 (1.8)	4 (5.3)
No	164 (93.5)	63 (95.5)	34 (89.5)	375 (97.2)	112 (98.2)	72 (94.7)
HADS-D T2 (mean ± SD)	2.46 (2.53)	3.27 (3.13)	3.75 (2.89)	2.23 (2.65)	2.27 (2.46)	2.70 (2.95)
HADS-D ≥ 8 T2						
Yes	11 (6.3)	10 (15.2)	5 (13.2)	20 (5.2)	6 (5.3)	6 (7.9)
No	164 (93.7)	56 (84.8)	33 (86.8)	366 (94.8)	108 (94.7)	70 (92.1)

Overall prevalence rates of anxiety symptoms (HADS-A ≥ 8) for women were 14.7% (*n* = 41) at time 1 and 14.0% (*n* = 39) at time 2. For men, corresponding prevalence rates were 6.6% (*n* = 38) at time 1 and 7.1% (*n* = 41) at time 2. Prevalence of depression symptoms (HADS-D ≥ 8) for women were 6.5% (*n* = 18) at time 1 and 9.3% (*n* = 26) at time 2. For men, corresponding prevalence rates were 3.0% (*n* = 17) at time 1 and 5.6% (*n* = 32) at time 2.

### Longitudinal Association of Change in PA With Anxiety

For women, there was a stable HADS-A score between time 1 (M = 3.95, SD = 3.65) and time 2 (M = 3.96, SD = 3.57, 95% CI; −0.28, 0.25, *t*(278) = −0.84, *p* = 0.93, Cohen’s *d* = 0.01. For men, HADS-A score increased from time 1 (M = 2.70, SD = 2.66) to time 2 (M = 2.93, SD = 2.91, 95% CI; −0.39, −0.07, *t*(575) = -2.80, *p* < 0.01, Cohens *d* = 0.12).

As displayed in Table 2, a binary logistic regression analysis did not support sex differences in the association between change in PA and anxiety, and test for statistical interaction (sex∗change in PA) was not statistically significant. For the whole sample, Increased PA (OR; 2.42, 95% CI, 1.10, 5.34, *p* < 0.05) was associated with increased risk of anxiety. In addition, baseline HADS-A (OR; 1.80, 95% CI, 1.60, 2.03, *p* < 0.001) and lower age (OR; 0.97, 95% CI, 0.94, 1.00, *p* < 0.05) were associated with higher risk for anxiety.

**TABLE 2:** Binary logistic regression of the associations between change in physical activity with anxiety (HADS-A **≥** 8) measured at time 2 (The fitness and mental health study, Norway, 2020–2021).

	HADS-A ≥ 8 T2					
	Total (*N* = 855		Women (*n* = 279)		Men (*n* = 576)	
	*N*	OR (95%CI)	*n*	OR (95%CI)	*n*	OR (95%CI)
Change in PA						
Unchanged	561	Ref.	175	Ref.	386	Ref.
Increased	180	2.42^a^ (1.10, 5.34)	66	1.90 (0.54, 6.67)	114	2.93 (0.99 8.69)
Reduced	114	1.60 (0.67, 3.82)	38	2.36 (0.61, 9.08)	76	1.05 (0.31, 3.52)

a Indicates statistically significant association at *p* < 0.05.

Note: PA, Physical activity; T2, timepoint 2. Adjusted for age, alcohol use, education, psychotropic drug, limiting long term illness and history of mental disorder. Total sample also adjusted for sex.

A linear regression analysis was conducted with HADS-A total score at time 2 as the dependent variable (Supplementary Table S2). Change in PA was not statistically significantly related to HADS-A score. For the sample as a whole, only baseline HADS-A score (*p* < 0.001) and limiting long-term illness (*p* < 0.05) were associated with increased anxiety levels at time 2, whereas higher age was associated with decreased anxiety levels (*p* < 0.05).

### Longitudinal Association of Change in PA With Depression

For women, mean HADS-D score increased from time 1 (M = 2.37, SD = 2.43) to time 2 (M = 2.83, SD = 2.77, 95% CI; −0.73, −0.19, *t*(278) = −3.33, *p* < 0.001, Cohen’s *d* = 0.20. For men, mean HADS-D score increased from time 1 (M = 1.99, SD = 2.23) to time 2 (M = 2.30, SD = 2.66, 95% CI; −0.47, −0.15, *t*(575) = −3.73, *p* < 0.001, Cohens *d* = 0.16).

A binary logistic regression analysis showed that compared to the Unchanged PA subgroup, Increased PA was associated with an increased risk for depression, odds ratio (OR); 2.40, 95% confidence interval (CI), 1.09–5.27, *p* < 0.01 for the complete sample (Table 3), but our test for interaction (sex∗change in PA) supported that this association was statistically different between men and women. For women who increased PA compared to the Unchanged PA subgroup, there was an increased risk of depression (OR; 7.34; 95% CI, 1.96–27.42, *p* < 0.01). In addition, the control variable baseline HADS-D score was significantly associated with risk of depression (OR; 1.92; 95% CI, 1.67–2.21, *p* < 0.001). For men, the change in PA was not significantly related to risk of depression, but the control variable baseline HADS-D score was (OR; 2.01; 95% CI, 1.65–2.44, *p* < 0.001).

**TABLE 3:** Binary logistic regression of the associations between change in PA with depression (HADS-D **≥** 8) measured at time 2 (The fitness and mental health study, Norway, 2020–2021).

	HADS-D ≥ 8 T2					
	Total (*N* = 855)		Women (*n* = 279)		Men (*n* = 579)	
	*n*	OR (95%CI)	*n*	OR (95%CI)	*n*	OR (95%CI)
Change in PA						
Unchanged	561	Ref.	175	Ref.	386	Ref.
Increased	180	2.40^a^ (1.09, 5.27)	66	7.34^b^ (1.96, 27.42)	114	1.03 (0.32, 3.37)
Reduced	114	1.05 (0.40, 2.71)	38	2.10 (0.42, 10.42)	76	0.60 (0.17, 2.20)

a Indicates statistically significant association at *p* < 0.05.

b Indicates statistically significant association at *p* < 0.01.

Note. PA, Physical activity. T2, timepoint 2. Adjusted for age, alcohol use, education, psychotropic drug, limiting long term illness and history of mental disorder. Total sample also adjusted for sex.

A linear regression analysis with HADS-D total score as the dependent variable showed that for women, Increased PA (*p* < 0.01), Reduced PA (*p* < 0.05) and baseline HADS-D score (*p* < 0.001) were associated with higher levels of depressive symptoms at time 2 (Supplementary Table S3). For men, Change in PA was not significantly related to depression symptoms. Of the control variables, baseline HADS-D score (*p* < 0.001) and use of psychotropic medication (*p* < 0.01) and lower age (*p* < 0.05) were significantly associated with higher levels of depressive symptoms at time 2.

## Discussion

The aim of the current study was to investigate if change in physical activity (PA) during the early phase of the COVID-19 pandemic predicted symptoms of anxiety and depression 6 months later in a sample of physically active adults. For women level of anxiety symptoms did not change from time 1 to time 2, whereas for men anxiety symptoms increased during the six-month period. We found an increase in level of depression symptoms in both women and men from baseline to time 2. Furthermore, we did not find support for sex differences in the association between change in PA and anxiety, however, for the total sample increased PA predicted increased risk for anxiety. Increased level of PA predicted a higher risk for depression for women, but not for men.

The findings that increased level of PA during the early phase of the COVID-19 predicted increased risk for anxiety symptoms for both women and men and higher risk for depressive symptoms for women were unexpected. The results may seem counterintuitive given the large body of research which shows that physical activity protects against mental health problems, including during the COVID-19 pandemic [11, 12, 18]. However, as evident from Figures 1, 2, the Reduced PA subgroup had relatively higher level of depressive symptoms compared to the Unchanged and Increased PA subgroups, and all PA subgroups except for men in the Reduced PA subgroup and women in the Unchanged PA subgroup had a slight increase in depression symptoms from time 1 to time 2. The largest increase in depression symptoms was in the women Increased PA subgroup, who reported an increase in depression symptoms from HADS-D = 2.14 (*SD* = 2.24) at time 1 to HADS-D = 3.31 (*SD* = 3.15) at time 2. Thus, it appears that even though the Increased PA subgroups had the highest increase in depression symptom level in comparison with the other subgroups, Reduced PA subgroups in both women and men had the highest relative level of depression symptoms compared to the other subgroups. In the logistic regression analysis, higher initial symptom level of both anxiety and depression symptoms was a significant control variable for severity of symptoms at time 2 for both women and men. This finding was consistent for measures of anxiety and depression. This means that individuals already experiencing heightened level of symptoms are at higher risk for further deterioration of mental health during the pandemic also among physically active persons.

The high level of physical activity in the sample must also be taken into account when interpreting the association between increased PA and risk of mental health symptoms. The majority of the respondents exercised more than the weekly amount recommended by the WHO. Studies show a huge variation in prevalence of adherence to the WHO physical activity guidelines in Norway and Europe [23], and the present sample is considerably more physically active than the general population. This implies that findings from the general public which show a protective effect of increased PA on mental health may not be directly transferable to populations who are characterized by high levels of PA. The homogenous nature of the present sample in terms of activity level may thus partly explain the unexpected association between anxiety and depression symptoms and PA level. Another possible explanation for the association between increased PA and symptoms of anxiety and depression may be that participants who experienced an increase in symptoms of anxiety and depression during the early stages of the pandemic increased their level of physical activity to better cope with mental health issues. The unexpected findings of this study may thus be the result of reverse causation, however, the observational design of the study precludes any conclusion regarding the causal direction.

In the present sample 9.2% had a HADS-A score indicating caseness of an anxiety disorder at time 1 and 9.1% at time 2. For HADS-D 4.3% met the cutoff criteria at time 1 and 6.9% at time 2. The results thus indicate a stability in anxiety symptoms and an increase in depression symptoms. However, the prevalence rates in the total sample are considerably lower than the 14.2% reporting anxiety and 9.4% reporting depression in the Norwegian general population during the pre-pandemic period [34]. Thus, even though there was an overall increase in depression symptoms from time 1 to time 2 in the present study, the sample had considerably lower rates of clinical levels of both anxiety and depression symptoms compared to the general population. The overall impression is therefore that the sample was characterized by important protective factors. In addition to the high physical activity level, education level was high, and consumption of alcohol was relatively low. Given these factors, which have been found to protect against mental illness, it is interesting to note that the sample nevertheless may have been influenced by the stressors posed by the pandemic situation. These results are in line with previous research that shows heightened anxiety both among healthy persons and individuals with pre-existing mental health problems during COVID-19 [35]. In addition, the results support previous research that found reductions in mental health and life satisfaction during confinements period of the pandemic among elite athletes and young athletes [25, 26].

It should be noted that there were considerable sex differences in the present sample with respect to level of anxiety and depression symptoms. As evident from Table 1, there was a higher proportion of women meeting the HADS cutoff for clinical symptom level of anxiety and depression across all PA subgroups, relative to men. Overall, women had stable levels of anxiety symptoms from time 1 to time 2, while men reported a small increase in anxiety symptoms. Still, anxiety level in women were higher than those of men both at time 1 and time 2. Even if women in the current study had lower levels of anxiety symptoms (14.7%) compared to women in the general population before the pandemic (17.4%) [34] the findings from the present study corroborate research during the COVID-19 which finds that women are more vulnerable to suffer from symptoms of mental problems compared to men despite being physical active [10]. A recent study on long-term incidence of anxiety among participants in an ultralong-distance cross-country ski race was associated with a lower risk of developing anxiety compared to adults from the general population [36]. However, in the latter study higher performance in women (measured as the finishing time to complete the race) was associated with increased risk of anxiety compared to slower skiing women. Thus, it is possible that anxiety symptoms drive certain persons to exert more extreme behaviors, and that this may be more pronounced in women who are physically active.

### Limitations

Although it is a strength that the current study includes a longitudinal survey of physical activity and mental health during the COVID-19 in physically active adults, it is a limitation that no pre-pandemic assessment was conducted. It is thus a risk that the participants’ level of anxiety and depression at time 1 was already heightened due to the pandemic situation compared to their pre-pandemic mental health state. Furthermore, women are underrepresented in the current sample, and given the well-known finding that women tend to report higher depressive and anxiety levels generally compared to men, the uneven gender distribution may have biased the results. The sample in the study were highly physically active and may therefore not necessarily be representable for adults with lower weekly exercise level. The second time of assessment was in January, during which the Norwegian climate is characterized by lack of daylight and low temperatures, and responses may reflect seasonal fluctuations in mood [37]. Fewer respondents participated at time 2 compared to time 1, and we cannot know if characteristics among those who did not respond could have influenced the results. For example it may be plausible that respondents with poorer mental health were less likely to respond. In addition, we have no information except for gender for those who were invited but did not participate at any time points, which means we cannot know if there were any systematic differences between the responders and non-responders. Finally, the use of self-report instruments to measure symptoms of anxiety and depression may lead to over-estimation of symptom severity, as a Norwegian study using clinical interviews did not find an increase in clinical levels of anxiety and depression during the COVID-19 pandemic [38]. A strength of the study is the longitudinal design, as there have been few long-term studies of physically active adults during the COVID-19.

In conclusion, the present study reports longitudinal results of anxiety and depression symptoms in a sample of physically active adults. The sample reported an increase in severity of depression symptoms over a six-month period, which was particularly true for women. Women and men who increased PA level in the initial phase of the pandemic had increased risk for anxiety, and women who increased PA had increased risk for depression, 6 months later. The study shows that the COVID-19 pandemic is associated with mental health problems also among individuals who adhere to recommendations for physical activity.
