# Supplementary material for: Does Change in Physical Activity During the Initial Phase of the COVID-19 Pandemic Predict Psychological Symptoms in Physically Active Adults? A Six-Month Longitudinal Study
Source: Int J Public Health. 2022 Jun 8;67:1604528. doi: 10.3389/ijph.2022.1604528 (PMC9216186; doi:10.3389/ijph.2022.1604528)
Supplement: Supplementary file 1 [file DataSheet1.docx]

**Supplementary material**

**Table S1.** Comparison between follow-up and lost to follow-up participants (The fitness and mental health study, Norway, 2020-2021)

|  | **Participated at time 1 and time 2**  **(*N* = 855)** |  | **Lost to follow-up**  **(*N* = 462)** |  | ***p-value*** |
| --- | --- | --- | --- | --- | --- |
|  | ***n* (%)** |  | ***n* (%)** |  |  |
| **Women** | 277 (32.4) |  | 130 (28.3) |  | .130^1^ |
| **Age, years** (mean ± SD) | 49.8 (11.3) |  | 47.5 (11.7) |  | .001^2^ |
| **Education**  Primary, secondary, tertiary  University < 4 years  University > 4 years | 71 (8.3)  279 (32.6)  505 (59.1) |  | 47 (10.2)  168 (36.5)  244 (53.0) |  | .120^1^ |
| **Lifetime history of mental disorder**  Yes  No | 99 (11.6)  756 (88.4) |  | 46 (10.0)  413 (89.8) |  | .329^1^ |
| **Limiting long term illness**  Yes  No | 91 (10.6)  764 (89.4) |  | 45 (9.8)  415 (90.2) |  | .676^1^ |
| **Current psychotropic drug**  Yes  No | 20 (2.3)  835 (97.7) |  | 7 (1.5)  452 (98.3) |  | .388^1^ |
| **Alcohol use**  Once per month or less  2-3 times per month  2-3 times per week  4 times per week or more | 255 (29.8)  310 (36.3)  241 (28.2)  49 (5.7) |  | 142 (30.9)  166 (36.1)  125 (27.2)  26 (5.7) |  | .973^1^ |
| **Current weekly physical activity**  Once a week or less  2-3 times a week  Almost daily | 10 (1.2)  210 (24.6)  633 (74.0) |  | 4 (0.9)  117 (25.4)  338 (73.5) |  | .834^1^ |
| **Change in physical activity**  Reduced  Unchanged  Increased | 114 (13.3)  561 (65.6)  180 (21.1) |  | 69 (15.0)  284 (61.7)  106 (23.0) |  | .430^1^ |
| **HADS-A** (mean ± SD) | 3.1 (3.1) |  | 3.3 (2.9) |  | .132^2^ |
| **HADS-A ≥ 8**  Yes  No | 79 (9.2)  776 (90.8) |  | 40 (8.7)  420 (91.3) |  | .800^1^ |
| **HADS-D** (mean ± SD) | 2.1 (2.3) |  | 2.3 (2.5) |  | .217^2^ |
| **HADS-D ≥ 8**  Yes  No | 35 (4.1)  820 (95.9) |  | 24 (5.2)  436 (94.8) |  | .294^1^ |

*Note*. HADS-A = Hospital Anxiety and Depression Scale Anxiety subscale. HADS-D = Hospital Anxiety and Depression Scale Depression subscale. ^1^Chi square. ^2^*t*-test. Cell counts may not add up to 100% due to missing values.

**Table S2.** Unstandardized beta (B) and 95% confidence intervals (CI) for the associations of change in PA on HADS-A total score measured at time 2 (The fitness and mental health study, Norway, 2020-2021)

|  | **HADS-A score T2** | | | | | | | | | | | | |
| --- | --- | --- | --- | --- | --- | --- | --- | --- | --- | --- | --- | --- | --- |
|  | **Total (*N* = 855)** | | | |  | | **Women (*n* = 279)** | | |  | **Men (*n* = 579)** | | |
|  | ***n*** | **B (95% CI)** | **Adj *R*^2^** | | |  | ***n*** | **B (95% CI)** | **Adj *R*^2^** |  | ***n*** | **B (95% CI)** | **Adj *R*^2^** |
| **Change in PA** |  |  | |  | |  |  |  |  |  |  |  |  |
| Unchanged | 561 | Ref. | | 0.62 | |  | 175 | Ref. | 0.67 |  | 386 | Ref. | 0.59 |
| Increased | 180 | 0.19 (-0.15, 0.53) | |  | |  | 66 | 0.50 (-0.11, 1.11) |  |  | 114 | 0.01 (-0.40, 0.41) |  |
| Reduced | 114 | -0.07 (-0.47, 0.33) | |  | |  | 38 | 0.36 (-0.40, 1.13) |  |  | 76 | -0.32 (-0.80, 0.16) |  |

*Note*. PA = Physical activity. T2 = timepoint 2. Adjusted for age, alcohol use, education, psychotropic drug, limiting long term illness, and history of mental disorder. Total sample also adjusted for sex.

| **Table S3.** Unstandardized beta (B) and 95% confidence intervals (CI) for the associations of change in PA on HADS-D total score measured at time 2 (The fitness and mental health study, Norway, 2020-2021) | | | | | | | | | | | |
| --- | --- | --- | --- | --- | --- | --- | --- | --- | --- | --- | --- |
|  |  | **HADS-D score T2** | | | | | | | | | |
|  | **Total (*N* = 855)** | | |  | **Women (*n* = 279)** | | |  | **Men (*n* = 579)** | | |
|  | ***n*** | **B (95% CI)** | **Adj *R*^2^** |  | ***n*** | **B (95% CI)** | **Adj *R*^2^** |  | ***n*** | **B (95% CI)** | **Adj *R*^2^** |
| **Change in PA** |  |  |  |  |  |  |  |  |  |  |  |
| Unchanged | 561 | Ref. | 0.44 |  | 175 | Ref. | 0.40 |  | 386 | Ref. | 0.48 |
| Increased | 180 | 0.28 (-0.07, 0.62) |  |  | 66 | .92^a^ (0.31, 1.54) |  |  | 114 | -0.11 (-0.52, 0.31) |  |
| Reduced | 114 | 0.12 (-0.30, 0.53) |  |  | 38 | .82^b^ (0.05, 1.60) |  |  | 76 | -0.21 (-0.69, 0.27) |  |

*Note*. PA = Physical activity. T2 = timepoint 2. Adjusted for age, alcohol use, education, psychotropic drug, limiting long term illness and history of mental disorder. Total sample also adjusted for sex. ^a^ indicates statistically significant association at *p* < .01. ^b^ Indicates statistically significant association at *p* < .05.
